# Unfolding Electrolyzer Characteristics to Reveal Solar‐to‐Chemical Efficiency Potential: Rapid Analysis Method Bridging Electrochemistry and Photovoltaics

**DOI:** 10.1002/cssc.202402027

**Published:** 2024-11-26

**Authors:** Oleksandr Astakhov, Thérèse Cibaka, Lars Wieprecht, Uwe Rau, Tsvetelina Merdzhanova

**Affiliations:** ^1^ Photovoltaics (IMD-3) Forschungszentrum Jülich GmbH 52425 Jülich Germany; ^2^ Faculty of Electrical Engineering and Information Technology RWTH Aachen University Mies-van-der-Rohe-Straße 15 52074 Aachen Germany

**Keywords:** Electrochemistry, Sustainable Chemistry, Solar fuels, Solar-to-chemical efficiency, Solar-to-fuel efficiency

## Abstract

Development of photovoltaic−electrochemical (PV‐EC) systems for energy storage and industry decarbonization requires multidisciplinary collaborative efforts of different research groups from both photovoltaic and electrochemical research communities. Consequently, the evaluation of the solar‐to‐chemical or solar‐to‐fuel efficiency of a new electrolyzer (EC) as a part of a PV‐EC system is a time‐consuming task that is challenging in a routine optimization loop. To address this issue, a new rapid assessment method is proposed. This method employs power balance requirements to unfold the input EC characteristics into the parameter space of PV‐EC systems. The system parameters, composed with the EC output characteristics, yield the solar‐to‐chemical efficiency attainable by the electrolyzer in combination with any PV device under any irradiance at any relative PV‐to‐EC scaling and any mode of power coupling. This comprehensive overview is achieved via a mathematically simple conversion of the EC characteristics in any spreadsheet software. The method, designed to streamline the development and minimize the efforts of both the photovoltaic and electrochemical communities, is demonstrated via the analysis of CO_2_‐reduction electrolyzer characteristics and verified with dedicated PV‐EC experiments.

## Graphical abstract

A method of unfolding current‐voltage characteristics of electrochemical (EC) cells to assess solar‐to‐chemical efficiencies achievable in combination with any photovoltaic (PV) device under any irradiance, PV‐to‐EC scaling, and power coupling mode. A comprehensive overview is achieved through a mathematically simple conversion of the EC characteristics in any spreadsheet software.

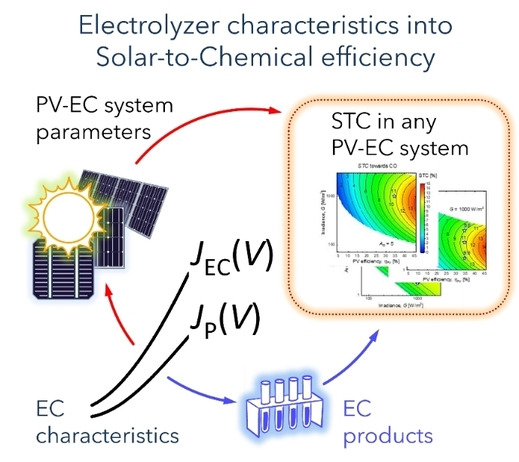

## Introduction

Combinations of photovoltaics (PV) with electrochemical (EC) devices represent a promising solution for solar energy storage and industry decarbonization. PV‐EC systems can produce hydrogen,[^1^, ^2^, ^3^, ^4^, ^5^, ^6^, ^7^, ^8^, ^9^, ^10^, ^11^, ^12^, ^13^, ^14^, ^15^, ^16^, ^17^] hydrocarbons via electrochemical CO_2_ reduction,[^18^, ^19^, ^20^, ^21^] fertilizers,[^22^, ^23^, ^24^, ^25^] or decontaminate water and soil.^[26]^ Concepts and designs of PV‐EC systems are addressed at different scales with a variety of PV devices (cells, modules, strings, arrays, fields) coupled to a variety of EC devices (electrochemical cells, electrolyzer stacks, electrolyzer modules). The most efficient laboratory‐scale PV‐EC devices are based on direct coupling,[^1^, ^2^, ^3^, ^4^, ^5^, ^6^, ^8^, ^9^, ^10^, ^11^, ^12^, ^13^, ^14^, ^15^, ^16^, ^17^, ^19^] whereas large‐scale field systems are often coupled using power electronics.[^7^, ^11^] These systems can produce single products such as hydrogen or multiple products such as CO, formic acid, and a variety of other chemicals.[^18^, ^19^, ^20^, ^21^, ^27^] Therefore variety of names such as “solar‐to‐hydrogen efficiency”,[^1^, ^2^, ^3^, ^4^, ^5^, ^6^, ^7^, ^8^, ^9^, ^10^, ^11^, ^12^, ^13^, ^14^, ^15^, ^16^] “solar‐to‐fuel efficiency”,[^17^, ^19^, ^20^, ^21^, ^25^] “solar‐to‐chemical efficiency”,[^18^, ^28^, ^29^] or “solar‐to‐product efficiency”,^[30]^ refer to the PV‐EC system energy efficiency. In this work we use “solar‐to‐chemical efficiency” (*STC*) as one of the most general terms.

The development of PV‐EC systems requires interdisciplinary collaborative efforts of different research groups from both photovoltaic and electrochemical research communities. Because of the multidisciplinary nature of the development, testing a new electrolyzer with a chosen PV device to evaluate the solar‐to‐chemical efficiency potential can be a difficult and time‐consuming task. To reduce the effort and facilitate the primary optimization of PV‐EC systems, it would be beneficial to have a quick method to assess the *STC* potential of an electrolyzer in various PV‐EC systems prior to laboratory or field testing. Such predictions are commonly made through numerical modeling,[^10^, ^11^, ^31^, ^32^, ^33^, ^34^, ^35^, ^36^, ^37^, ^38^] however, the complexity of the models and simulation methods makes it difficult for routine practical application. Typical equations describing both the PV[^39^, ^40^, ^41^] and EC[^42^, ^43^, ^44^] parts of the system require iterative numerical solution methods and fitting to the experimental data.^[45]^

This paper presents an alternative, mathematically simple method for assessing the solar‐to‐chemical efficiency that an electrolyzer can achieve in combination with photovoltaics of any efficiency and scale under any irradiance and mode of coupling. Rather than iteratively optimizing a specific PV‐EC system around the electrolyzer in question, we propose to reverse the logic of the analysis and address the entire set of possible optimal PV‐EC systems, which remarkably does not require system simulations. Since every optimal PV‐EC system drives the electrolyzer at some operating point on its current density‐voltage characteristic *J*_EC_(*V*), every point of this *J*_EC_(*V*) corresponds to a set of optimal PV‐EC systems. We can use power balance in PV‐EC system to determine *what system parameters are required at any EC operating point*. In this way, the input characteristic of the electrolyzer is “unfolded” into the parameter space of optimal PV‐EC systems that can potentially be built around this electrolyzer. Concurrently, the chemical product output of the electrolyzer at each operating point is given by its product current density‐voltage characteristic *J*_p_(*V*). Hence, for any given EC operating voltage, we can determine (i) the combination of required PV‐EC system parameters, such as irradiance, PV efficiency, and PV‐to‐EC area ratio, and (ii) the product output power. Irradiance, area ratio, and product power are sufficient to calculate the STC value. This operation, applied to the entire set of measured EC operating voltages, yields two sets of key quantities: first, the set of optimal PV‐EC system parameter combinations, and second, the set of *STC* values that these systems would have. The pairwise correspondence of the system parameters and *STC* values at each EC operating voltage leads to the desired dependencies that describe what *STC* the analyzed electrolyzer would achieve in combination with any PV device under any irradiance and at any relative PV‐to‐EC scaling. This information is obtained without system modelling through a mathematically simple transformation of the electrolyzer characteristics, which can be performed in any automatic spreadsheet software. In our previous work, we presented the first key principle of reverse analysis applied to assess the solar‐to‐hydrogen efficiency limit in PV‐EC water splitting systems.^[46]^ In this work, we generalize the method for treating electrolyzers with an arbitrary number of products, nonideal Faradaic efficiencies, and various PV‐EC system parameters, including coupling efficiency. The method is applied to a direct‐coupled PV‐EC system in this study, but it is equally applicable to systems with power conditioning electronics between the PV and EC system parts.

In this paper, we first present the general logic of reverse analysis and formulate how essential PV‐EC power balance equations are used to convert the EC characteristic into desired quantities. The method is then applied to analyze the characteristics of an experimental CO_2_ reduction reaction (CO_2_RR) EC cell with a Ag catalyst, which produces CO as the main product and H_2_ as a byproduct. This common CO_2_‐to‐CO electrolysis^[47]^ is chosen to demonstrate the analysis of a general multiproduct case with a minimal number of products. Finally, we verify the results of the analysis by testing the analyzed electrolyzer in combination with high‐efficiency concentrator GaAs solar cells.

Given the wealth of information revealed by the mathematically simple procedure, we believe the method will be helpful to streamline PV‐EC development and minimize the efforts of both electrochemical and photovoltaic communities. To facilitate implementation of the method, we prepared an example spreadsheet with reverse analysis as supplementary material.

## Generic PV‐EC System and Power Balance

**Figure 1:**
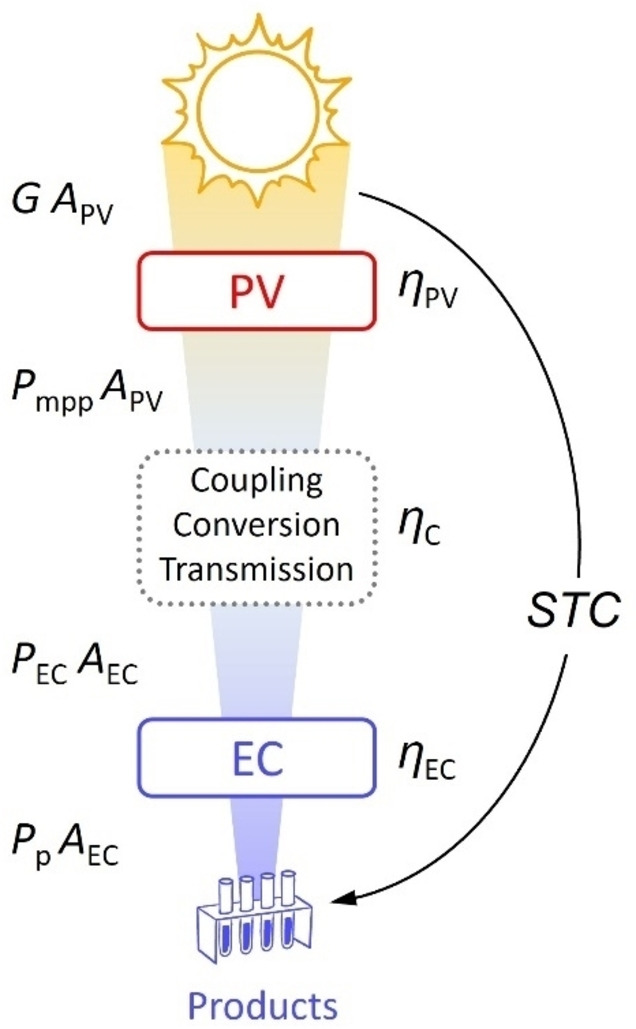
Block diagram of a generalized PV‐EC system.

The reverse analysis method is based on the power balance in a PV‐EC system, which we can formulate considering a generalized PV‐EC system shown schematically with a block diagram in Figure 1. This system converts sunlight into chemical products in three main steps. First, sunlight is converted into electricity with a PV device (cell, module, string, etc.); second, this electricity is transferred to an EC device via a coupling medium (directly via contact interfaces, plates, wires, or indirectly via a DC‐DC converter, inverter, or power grid); and finally, the electricity is used in the EC device (electrochemical cell, electrolyzer stack, electrolyzer module) to produce the desired chemical products.

The straightforward power flow in the generic PV‐EC system depicted in Figure 1 has one noteworthy key aspect. All powers in Figure 1 are expressed as products of the power densities and areas of the respective elements. All quantities designated as “*P*” with various subscripts refer to the power densities of either EC or PV. This is because the goal is to identify the solar‐to‐chemical efficiency potential of an electrolyzer based on its fundamental current density‐voltage characteristics. Electrolyzers can produce single or multiple products with varying faradaic and voltage efficiencies. It is more convenient to evaluate the performance of a PV‐EC system and calculate *STC* towards each product individually. Therefore, the power density *P*_p_ in Figure 1 and throughout the text refers to one specific product in question. Another benefit of treating the problem via power densities is that the PV‐to‐EC area ratio *A*_R_, which is a fundamental system design parameter,[^1^, ^3^, ^32^, ^37^, ^48^, ^49^, ^50^, ^51^, ^52^] is presented explicitly in the equations. The area ratio is defined as:

(1) $$A_{R}=\frac{A_{PV}}{A_{EC}}\;$$

where *A*_PV_ is the geometric aperture area of the PV device (aperture area of the concentrator lens or mirror array for the case of concentrator PV), and *A*_EC_ is the total geometrical EC area (total area of all cells in the case of an electrolyzer stack).

The power flow block diagram in Figure 1 is sufficient to define the key power balance equations and link the power densities and efficiencies with the *STC* in a generic PV‐EC system. In the first step of the energy conversion chain in Figure 1 the solar irradiance *G* is converted by the PV device into electricity with a maximal power density *P*_mpp_ (mpp – maximum power point) at the PV efficiency *η*_PV_ :

(2) $$\eta_{PV}=\frac{P_{mpp}}{G}\;$$

During realistic operation, the PV actual working point may deviate from the maximum power point, resulting in power mismatch or “coupling loss”.[^10^, ^11^, ^37^, ^53^, ^54^, ^55^] The transmission of power to the EC device in direct‐coupled systems[^1^, ^2^, ^3^, ^4^, ^5^, ^6^, ^8^, ^9^, ^10^, ^11^, ^12^, ^13^, ^14^, ^18^, ^19^, ^55^] or current‐voltage conversion in DC‐DC‐ or inverter‐coupled systems[^7^, ^11^] may cause additional power losses. We lump all coupling conversion and transmission losses into a coupling efficiency *η*_C_, defined as:

(3) $$\eta_{C}=\frac{A_{EC}P_{EC}}{A_{PV}P_{mpp}}=\frac{P_{EC}}{A_{R}P_{mpp}}\;$$

At the transition between the PV and EC parts of the system, the area ratio must be considered because the power collected over the PV device area is transferred and distributed over the EC device area. In the next step, the EC converts its input electrical power density *P*_EC_ into one or more products, each with the product power density *P*_p_. The EC efficiency towards each individual product is given by:

(4) $$\eta_{EC}=\frac{P_{p}}{P_{EC}}\;$$

Overall, the system converts the input irradiance *G* arriving at the PV of area *A*_PV_ into the output product power density *P*_p_ collected over the electrolyzer area *A*_EC_ with a solar‐to‐chemical efficiency *STC*:

(5) $$STC=\frac{A_{EC}P_{p}}{A_{PV}G}=\frac{P_{p}}{A_{R}G}\;$$

This logical link is represented by the curved arrow in Figure 1. At the same time, we can express *STC* as the product of the efficiencies of the conversion steps in the power conversion chain:

(6) $$STC=\eta_{PV}\;\eta_{C}{\;\eta }_{EC}\;$$

Because *P*_p_ in Figure 1 and in equations (4) and (5) represents the power density of a single EC product, the *STC* represents the solar‐to‐chemical efficiency towards the single product. If an electrolyzer produces multiple products, each product can be analyzed separately, and the total *STC* can be calculated as the sum of the individual product *STC*’s if necessary. The core power balance between PV and EC parts of the system in power density units is:

(7) $${{\;\eta }_{PV}GA}_{R}\eta_{C}=P_{EC}\;$$

The presented set of equations links the EC input power density *P*_EC_ and output power density *P*_p_ to three key PV‐EC design parameters: PV efficiency *η*_PV_ (type of PV device), irradiance *G* (system′s input power density), and the area ratio *A*_R_ (mutual PV‐EC scaling). We use these equations for the analysis presented in the next section. Note that all the power balance equations presented above and their modified forms in the subsequent sections do not include or require current or voltage matching between the PV and EC devices, only power matching. Therefore, this analysis is equally applicable to direct‐coupled systems and systems with power‐matching electronics of any type, provided that the coupling efficiency *η*_C_ is properly treated.

## Principle of the Analysis

The principle of analysis is best presented, starting with the definition of the problem. Consider an EC device with a total area *A*_EC_ characterized by its input current density‐voltage characteristics *J*_EC_(*V*) and output product characteristics *J*_p_(*V*). These two characteristics include Faradaic efficiency, voltage efficiency, and total power conversion efficiency towards the specific product. Given these characteristics, *what solar‐to‐chemical efficiency can this EC achieve as a part of a PV‐EC system?* The answer to this question is represented by a set of solutions because a variety of PV devices of different sizes under different irradiances can be coupled to the EC device in different ways. Each solution in the set is a combination of the system parameters with the corresponding *STC* achievable by the system. The minimal set of PV‐EC system parameters includes the PV efficiency *η*_PV_, irradiance *G*, and PV‐to‐EC area ratio *A*_R_. To assess the maximum/limit of STC attainable by the electrolyzer, we assume a PV‐EC system with no coupling losses (*η*_C_=1). Since *every optimal PV‐EC system drives the electrolyzer at some operating point on the J*_EC_(*V), each point on the J*_EC_(*V*) *corresponds to a subset of optimal systems with a tractable combination of* (*η*_PV_, *G*, *A*_R_). We propose to access the entire set of (*η*_PV_, *G*, *A*_R_) combinations via the power balance equation (7) by finding the combinations of system parameters that match *the P*_EC_ at each operating voltage on the input *J*_EC_ *(V)* characteristics. At the same time, for each operating voltage, the product output *J*_p_(*V*) characteristic gives the product power density *P*_p_. Once *η*_PV_, *G*, *A*_R_, and *P*_p_ are defined, equation (5) yields the *STC*. By repeating this operation for each point of *J*_EC_(*V*) and *J*_p_(*V*), we unfold the EC characteristics into a four‐dimensional space of *η*_PV_, *G*, *A*_R_, and *STC*, as described in detail below. Thereby, we arrive at two corresponding data sets. The set of (*η*_PV_, *G*, *A*_R_) combinations and the set of corresponding *STC* values, which when taken together, represent the answer to the initial question for the entire range of PV‐EC operating conditions. Note that the problem is solved by the correspondence between the input and output EC power densities and the parameters derived from them using the power balance rule. In doing so, we can avoid considering and simulating the electrochemical processes that determine the EC characteristics *J*_EC_(*V*) and *J*_p_(*V*) and their relationship.

For practical application of the method, the power balance equations are adopted for the reverse analysis of the EC characteristics in the following way. At any voltage point of the EC characteristics the input EC power density is:

(8) $$P_{EC}\left(V\right)=VJ_{EC}\left(V\right)\;$$

where (*V* is the voltage of a single EC cell), and the output power density is
(9)

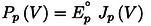




where *E* ^°^_p_ is the reference potential for the product in question (e. g. 1.23 V for H_2_, 1.34 V for CO, etc.).

The EC input power density is linked to the system parameter space via the power balance equation (7), which can be rearranged as:

(10) $$P_{EC}=A_{R}G\eta_{PV}\;\eta_{C}\;$$

We consider the coupling efficiency to be unity, but *η*_C_ is present in all equations and can be adjusted if necessary. For example, if the method is applied to a system with power conditioning between the PV and EC devices, the power overhead of the power electronics can be accommodated by modifying *η*_C_.

The link between the output EC power density and *STC* is already established by the equation (5) which can be rearranged for consistency:

(11) $$P_{p}=A_{R}G\;STC\;$$

Finally, combining (8) and (10) yields the link between the EC input JV characteristics and system parameters as:

(12) $$J_{EC}\left(V\right)=\frac{A_{R}G\eta_{PV}\;\eta_{C}}{V}\;$$

and the link between the EC output JV and *STC* is obtained by combining (9) and 11:
(13)

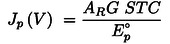




**Figure 2:**
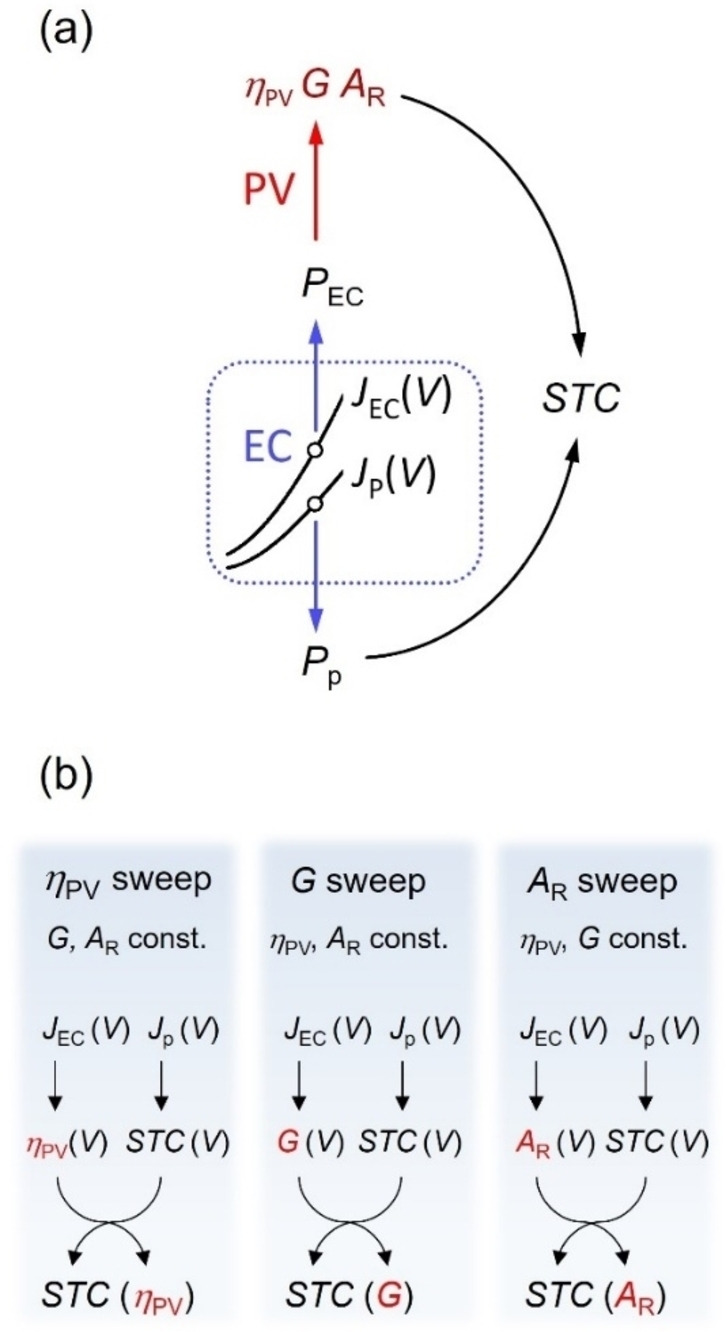
(a) An abstract presentation of a generic PV‐EC system in which the key quantities are represented by nodes and the direction of analysis is indicated by arrows. The analysis begins with the EC characteristics, which are highlighted by a dotted frame. (b) Reverse analysis algorithm to achieve dependencies of STC on the three key parameters *η*_PV_, *G*, and *A*_R_

To calculate the *STC*, we need the output power density, input power density, and area ratio, as shown by the black arrows in Figure 2(a). Therefore, the *STC* is determined after the system parameters are obtained. At any EC voltage, equation (12) links the EC input current density to a set of optimal PV‐EC systems described by *η*_PV_, *G*, and *A*_R_. At the same EC voltage scale, equation (13) links the EC output to the *STC*. Using equations (12) and (13), we unfold the EC characteristics into the parameter space of PV‐EC systems and access the hidden potential of the electrolyzer in terms of *STC*. The correspondence between combinations of (*η*_PV_, *G*, *A*_R_) and *STC* brings us to an implicit solution to the initially formulated problem – we determine what *STC* this electrolyzer can achieve as a part of any possible optimal PV‐EC system. Obviously, the subset of optimal (*η*_PV_, *G*, *A*_R_) parameter combinations satisfying the power balance requirement is infinite, even at a single EC operating point. The solutions of equations (12) and (13) constitute a four‐dimensional surface of finite area that encompasses the entire infinity of all possible parameter sets of optimal PV‐EC systems that can be built with the given electrolyzer and the corresponding *STC* values. The shape and boundaries of this surface are determined by the EC characteristics.

For practical applications, we first focus on two‐dimensional cross sections of this four‐dimensional surface, where only one parameter of *η*_PV_, *G* or *A*_R_ is free and “sweeps” along its axis to satisfy the power balance requirement, while the other two are assumed to be constant. This enables us to acquire dependencies of *STC*(*η*_PV_) in the “*η*_PV_ sweep,” *STC*(*G*) in the “*G* sweep” and *STC*(*A*_R_) in the “*A*_R_ sweep, ” as shown schematically in Figure 2(b).

Each column in Figure 2 (b) contains three analysis steps: (i) *J*_EC_(*V*) is converted into one of the *η*_PV_(*V*), *G*(*V*), or *A*_R_(*V*) dependencies; (ii) *J*_p_(*V*) with irradiance and area ratio is used to find *STC*(*V*); (iii) the free parameter is used as the x‐axis, and *STC* is used as the y‐axis to compose the *STC*(*η*_PV_), *STC* (*G*), and *STC* (*A*_R_) dependencies. These dependencies encompass the maximum *STC* that this electrolyzer can achieve with a PV device of any efficiency under any irradiance in a system with any PV‐to‐EC relative scaling.

For the *η*_PV_ sweep, we determine the PV efficiency required to drive the electrolyzer at any point of its *J*_EC_(*V*) under constant *G* and *A*_R_ yielded by rearranging equation (12) as:

(14) $$\eta_{PV}\left(V\right)=\frac{VJ_{EC}\left(V\right)\;}{A_{R}G\eta_{C}}\;$$

Rearranging equation (13) yields the *STC* at each point of *J*_p_(*V*) in the *η*_PV_ sweep:
(15)

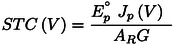




Having established the two dependencies, *η*_PV_(*V*) and *STC*(*V*), we complete the *η*_PV_ sweep by taking *η*_PV_ as the x‐axis and *STC* as the y‐axis and construct the dependence of *STC*(*η*_PV_), as shown schematically in the left column in Figure 2(b). The operation is equivalent to the unavailable algebraic inversion of *η*_PV_(*V*) dependence (14) to express voltage and substitute it in the STC dependence (15). The resulting composed dependence, *STC*(*η*_PV_), is tabular in form and contains the desired maximum of *STC* that this EC can reach in combination with a PV device of any efficiency for the assumed irradiance and area ratio.

The role of irradiance is studied in the “*G* sweep” shown schematically in Figure 2(b), with *G* as a free parameter while *η*_PV_ and *A*_R_ are held constant. To determine the irradiance required to drive the EC at each operating point of *J*_EC_(*V*), equation (12) is rearranged for constant *η*_PV_ and *A*_R_. This yields an irradiance *G* that satisfies the power balance at any EC voltage:

(16) $$G\left(V\right)=\frac{VJ_{EC}\left(V\right)\;}{A_{R}\eta_{PV}\eta_{C}}\;$$

For constant *η*_PV_ and *A*_R_, the voltage dependence of the *STC* towards an individual product is obtained by rearranging equation (13) and substituting *G* in with equation 16:
(17)

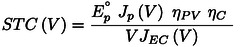




The second pair of desired dependencies, *G*(*V*) and *STC*(*V*), is now set. The dependence *STC*(*G*) is then constructed using the pairwise correspondence of *G* and *STC* values at each voltage, as shown in the middle pillar in Figure 2(b). This tabular dependence describes the maximum of *STC* that the EC can reach under any irradiance at the chosen *η*_PV_ and *A*_R_.

Finally, we consider the effect of the relative PV‐to‐EC scaling in the *A*_R_ sweep, as shown in the right column in Figure 2(b). The dependence of *A*_R_ satisfying the power balance in the system on the EC voltage is obtained by rearranging equation (12) as follows:

(18) $$A_{R}\left(V\right)=\frac{VJ_{EC}\left(V\right)\;}{G\eta_{PV}\eta_{C}}\;$$

Substituting *A*_R_ in equation (13) with equation (18) yields the desired dependence of *STC* towards an individual product on the EC voltage in the *A*_R_ sweep:
(19)

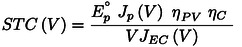




With the third pair of desired dependencies *A*_R_(*V*) and *STC*(*V*), we construct the final tabular dependence of *STC*(*A*_R_), as sketched in Figure 2(b).

Notably, the expressions for *STC* (17) and (19) converge to identical forms when *G*(V) and *A*_R_(V) are substituted with their respective equations. This is because the effects of *G* and *A*_R_ are equivalent. Both modulate the total input power available for PV conversion. In contrast, the expressions for the variable PV efficiency *η*_PV_(*V*) diverge because the total input power is constant, whereas the PV conversion efficiency varies.

A summary of the entire procedure for constructing the key dependencies *STC*(*η*_PV_), *STC*(*G*), and *STC*(*A*_R_) is presented in Table 1.

**Table 1:** Summary of the input characteristics, constants and equations used to obtain *STC* dependencies on *η*_PV_, *G*, and *A*_R_.

*Dependence*	*EC characteristics*	*Constants*	*x‐axis, equation*	*y‐axis, equation*
				
*STC*(*η*_PV_)	*J*_EC_(*V*), *J*_p_(*V*)	*G*, *A*_R_, *η*_C_, *E* ^°^_p_	*η*_PV_, (14)	*STC*, (15)
*STC*(*G*)	*J*_EC_(*V*), *J*_p_(*V*)	*η*_PV_, *A*_R_, *η*_C_, *E* ^°^_p_	*G*, (16)	*STC*, (17)
*STC*(*A*_R_)	*J*_EC_(*V*), *J*_p_(*V*)	*η*_PV_, *G*, *η*_C_, *E* ^°^_p_	*A*_R_, (18)	*STC*, (19)

It is evident that no simulations or physical models are employed in the procedure, which is a mathematically straightforward conversion of the EC characteristics. The set of potential systems is determined by the EC output, and we reveal or translate the information enveloped in the EC characteristics into the desired system parameters and *STC*. As a result of the unfolding of the EC characteristics, the dependencies *STC*(*η*_PV_), *STC*(*G*), and *STC*(*A*_R_) inherit the resolution and accuracy of the input data. If the desired value of *η*_PV_, *G*, or *A*_R_ is not present in the resulting dataset, it must be determined via interpolation between adjacent points. Given the relatively smooth behavior of the EC characteristics, we expect that linear interpolation will be an appropriate method in most cases. In return, these calculations can be applied to the experimental EC characteristics using any automatic spreadsheet software in a single step. Given the wealth of information revealed by the reverse analysis, we believe the method will be helpful to streamline PV‐EC development and minimize the efforts of both electrochemical and photovoltaic communities. To facilitate the application of the method, we prepared an example spreadsheet with analysis as a supplementary material.

## Application to CO_2_ Reduction Electrolyzer

**Figure 3:**
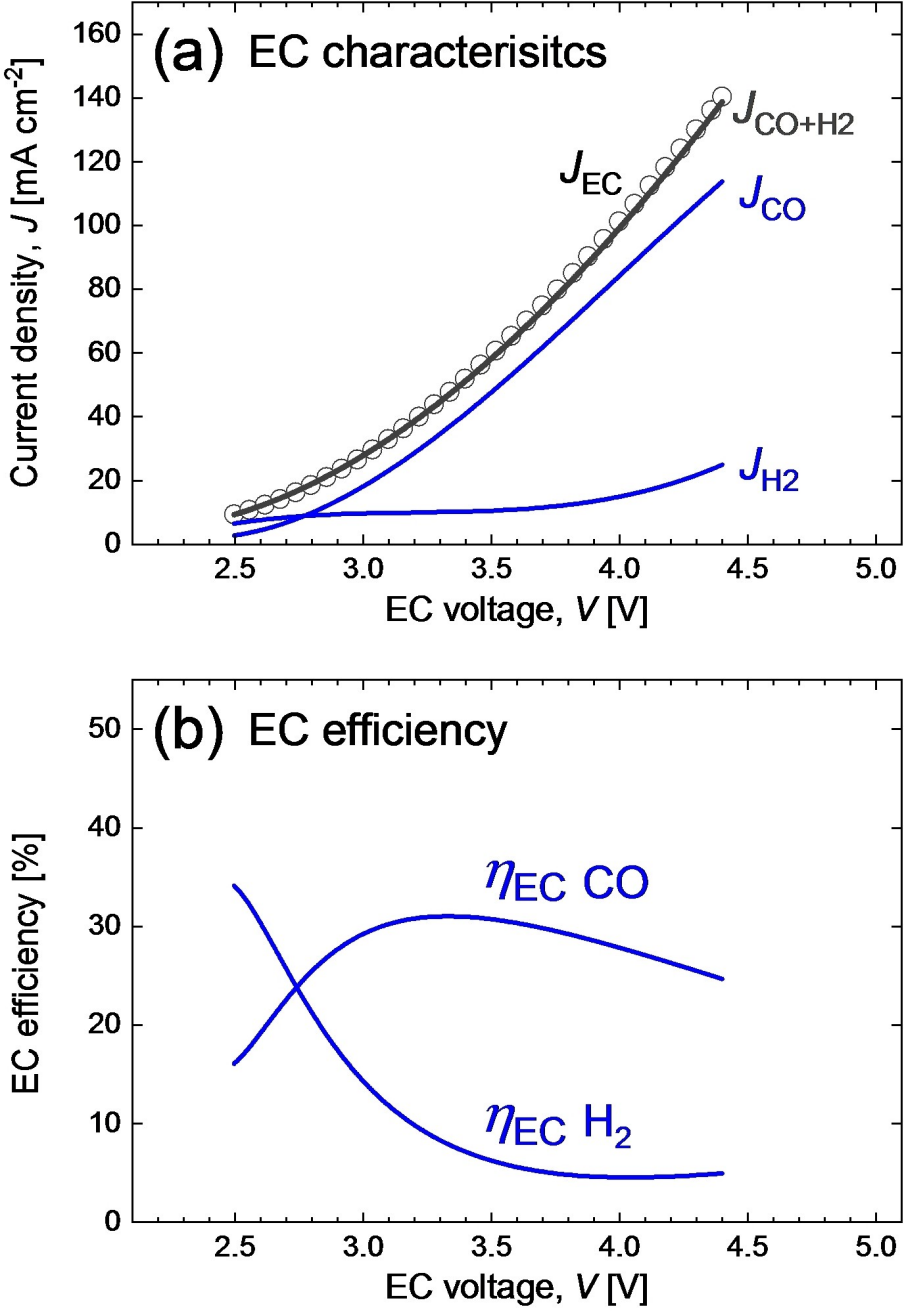
(a) EC characteristics: input current density *J*_EC_(V), output CO current density *J*_CO_(*V*), output H_2_ current density *J*_H2_(*V*), and total products current density *J*_CO+H2_(*V*) of the CO_2_RR electrolyzer. (b) EC efficiency *η*_EC_ towards individual products CO and H_2_

To demonstrate the practical use of this method, we apply the reverse analysis to our CO_2_RR EC cell with a Ag gas diffusion electrode, Nafion membrane and CO_2_ saturated 1 M KHCO_3_ electrolyte. The electrolyzer produces CO as the main target product and H_2_ as a byproduct in the CO_2_ reduction reaction. Therefore, for one input EC characteristic *J*_EC_(*V*), we have two output product characteristics: *J*_CO_(*V*) for CO and *J*_H2_(*V*) for H_2_. The sum of the product currents, *J*_CO_(*V*) and *J*_H2_(*V*), yields the total product current density *J*_CO+H2_(*V*). All three primary dependencies and the sum of the products presented in Figure 3(a) were approximated with polynomial fits to smooth out minor current fluctuations for the sake of presentation clarity. The smoothing procedure and original data are presented in the experimental section in Figure 8(a).

The electrolyzer demonstrates consistent performance without any noticeable Faradaic losses. The current density of the total products, *J*_CO+H2_(*V*), overlaps closely with the input current density, *J*_EC_(*V*). The total Faradaic efficiency and the Faradaic efficiencies towards both products are presented in the experimental section in Figure 8(b). Figure 3(b) presents the efficiency of the EC cell towards CO and H_2_ calculated according to equation (4) with *P*_EC_ determined by equation (8) and *P*_p_ by equation (9), using *E*°_CO_=1.34 V, and *E*°_H2_=1.23 V. The analysis does not require explicit treatment of EC efficiency; however, presentation of EC efficiencies can be revealing for understanding the results of the analysis and is relevant for cross‐comparison of results in the field.^[56]^ As shown in Figure 3, the electrolyzer exhibits high selectivity towards CO, which dominates the current above 3 V, while hydrogen production is suppressed. Figure 3(b) illustrates that with an increase in voltage from 2.5 V to approx. 3.1 V, the EC efficiency towards CO increases and a wide CO efficiency maximum of approx. 30 – 31 % is observed between 3.1 V and 3.6 V. Any combination of PV‐EC system parameters that drives EC into the latter voltage range is beneficial for the PV‐EC performance in terms of *STC*. A further increase in voltage results in a gradual reduction of efficiency due to increasing overpotential losses.

**Figure 4:**
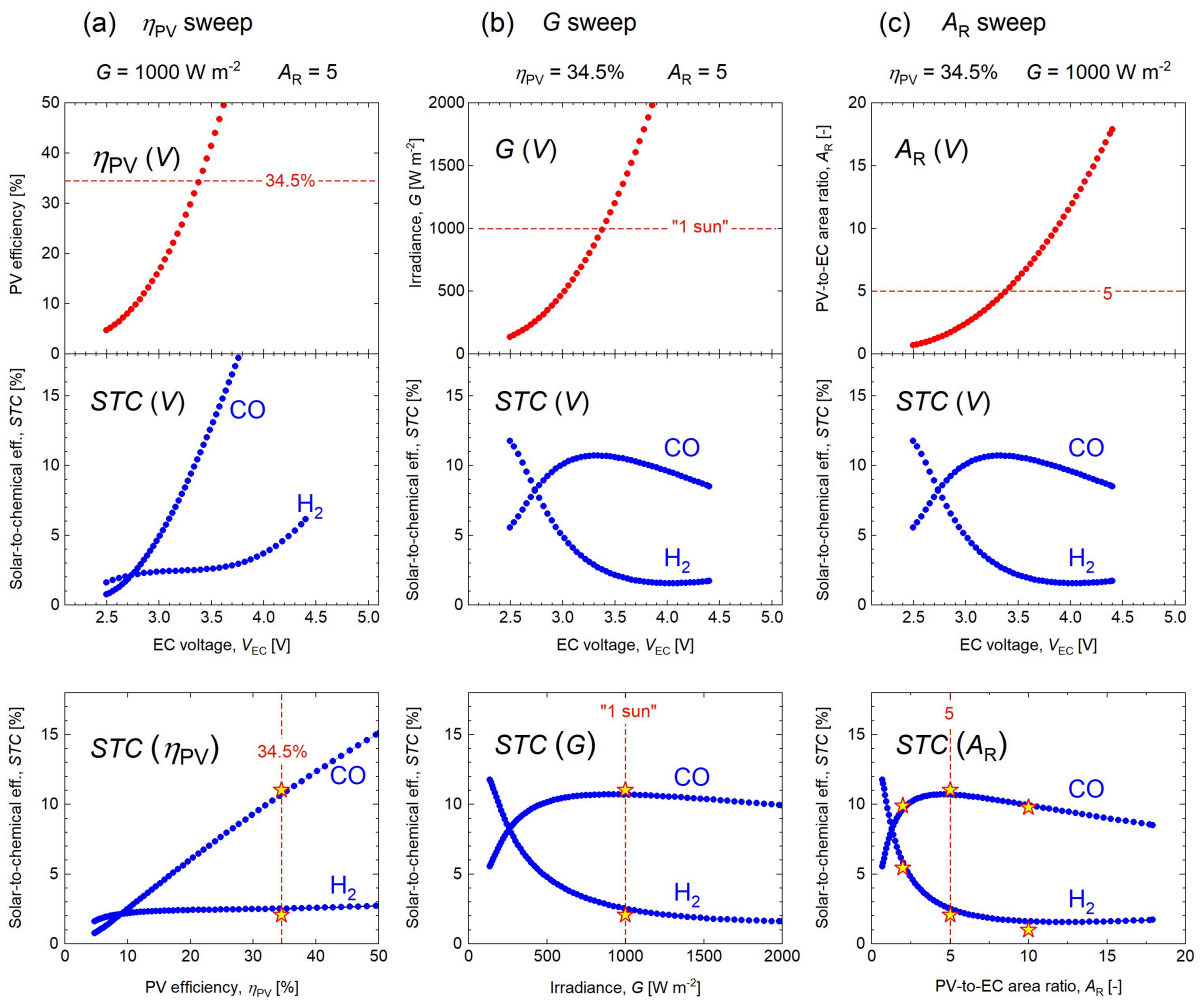
Stages and results of the reverse analysis applied to the EC characteristics presented in Figure 3(a) according to the algorithm summarized in Figure 2(b) and Table 1. Coupling efficiency is assumed to be *η*_C_=1, *E*°_CO_=1.34 V, *E*°_H2_=1.23 V, other constants are indicated in the graph panels. Stars and dashed lines represent the results of the PV‐EC experiment with emulated PV module with *η*_PV_=34.5 %.

The EC characteristics presented in Figure 3(a) are now translated into three key dependencies, *STC*(*η*_PV_), *STC*(*G*), and *STC*(*A*_R_), according to the analysis procedure summarized in Figure 2(b) and Table 1. The main stages of analysis and the resulting two‐dimensional slices of the solution surface are presented in Figure 4 in three columns of graphs for the *η*_PV_ sweep, *G* sweep, and *A*_R_ sweep, respectively.

The *η*_PV_ sweep is presented in Figure 4(a) for *G*=1000 W m^−2^, *A*_R_=5, *η*_C_=1, *E*°_CO_=1.34 V, and *E*°_H2_=1.23 V. At a fixed irradiance, as the electrolyzer power increases with voltage, the PV efficiency must also increase to maintain the power balance. This pushes up the maximal *STC* values for CO but also to some extent for H_2_, even though the selectivity towards H_2_ is heavily suppressed. The results of the *η*_PV_ sweep and the dependencies of *STC*(*η*_PV_) for both CO and H_2_ are plotted in the bottom graph in Figure 4(a). We can see that *STC* towards CO increases throughout the entire analyzed range, however the slope is sublinear. The trend is primarily dominated by the increase in PV efficiency. Even when the production of CO drops significantly, the increase in PV efficiency pulls *STC* values up. This is not the case for H_2_, whose production is strongly suppressed throughout most of the operating voltage range.

Even with high CO selectivity, the electrolyzer can reach 10 % *STC* towards CO only at PV efficiencies above 32 % (under *G*=1000 W m^−2^ and *A*_R_=5). This can be achieved in contemporary practice only with high end technologies such as multijunction III−V type PV or best examples of Perovskite‐Si tandems.^[57]^ The more common case of Si PV modules with *η*_PV_ of 20–23 % would result in *STC* of 6–7 % towards CO.

The predictions of the reverse analysis were cross‐checked with the experiment in which the same electrolyzer was connected to a PV device operating under a set of conditions selected from the analysis dataset. Three experiments were conducted with the same electrolyzer direct‐coupled to a high‐efficiency PV device with *η*_PV_=34.5 % for three area ratios (2, 5, and 10) under one sun, as described in the methods section. The measurements taken under *A*_R_=5 are presented in Figure 4(a) because the *η*_PV_ sweep was calculated with this area ratio. The stars in Figure 4(a) present the results of the experiment as *STC* towards both CO and H_2_. These results are in good agreement with the reverse analysis. In the PV‐EC experiment, the electrolyzer showed slightly higher selectivity towards CO than in the reference EC characterization run, resulting in a slightly higher *STC* with respect to the analysis predictions. This enhanced CO selectivity implies a further reduction in H_2_ evolution, which is consistently observed by the reduction of *STC* towards H_2_.

The results of the *G* sweep and *A*_R_ sweep are presented in Figure 4(b) and (c), respectively. In the case of *the G* sweep, the dependencies demonstrate the maximum *STC* that can be achieved by this electrolyzer in a PV‐EC system under various irradiance conditions, assuming a fixed *η*_PV_ and *A*_R_. In contrast to the dependence on PV efficiency in Figure 4(a), the irradiance dependence of *STC* towards CO exhibits a clear maximum in Figure 4(b). In the *G* sweep, the PV efficiency remains constant and *STC* is mostly determined by the EC efficiency across the entire operating power range. The EC efficiency towards CO has its peak in the middle of the operational range, as illustrated in Figure 3(b), and this peak is quantified in terms of *STC* in Figure 4(b). The position of this maximum can be varied by the area ratio or affected by the PV efficiency and must be matched to the target application irradiance for optimal system performance. The results of the PV‐EC experiment, represented by the stars in the bottom graph in Figure 4(b), are in good agreement with the reverse analysis.

The dependencies in Figure 4(c), calculated in the *A*_R_ sweep, have qualitative similarities with those calculated in the previous *G* sweep because the variations in area ratio as well as variations in irradiance both modulate the input power into the system (see Figure 1). Nevertheless, for practical PV‐EC optimization, it is instructive to quantify the effect of both *A*_R_ and *G* individually. The resulting dependence of *STC*(*A*_R_) calculated for the CO evolution in Figure 4(c) peaks at *A*_R_ ≈5. From the *STC*(*A*_R_) graph, it is evident that multiplication of the area ratio will result in a significant decrease in *STC*. Given that typical industrial water electrolyzers have *A*_R_ of 300–1500 or even higher, it is evident that the CO_2_RR electrolyzer used in the experiment must be further improved to shift the peak performance towards higher *A*_R_. The results of the PV‐EC experiment preformed for *A*_R_ of 2, 5, and 10 represented by the dashed lines and stars in Figure 4(c) are in excellent agreement with the predictions of the reverse analysis.

After examining the three dependencies of *STC* on *η*_PV_, *G*, and *A*_R_ individually, the next logical step is to investigate their interplay. The *STC* curves in the bottom panel of Figure 4 represent two‐dimensional slices of the four‐dimensional surface encompassing all possible *η*_PV_, *G*, *A*_R_, and *STC* combinations of all optimal PV‐EC systems that can potentially be constructed with the electrolyzer in question. We may now proceed to examine three‐dimensional slices, allowing two parameters to vary while the third is held constant. For example, we may calculate *STC* as a function of *η*_PV_ *and G* for a fixed *A*_R_ by performing the *η*_PV_ sweep iteratively for a range of irradiance values.

**Figure 5:**
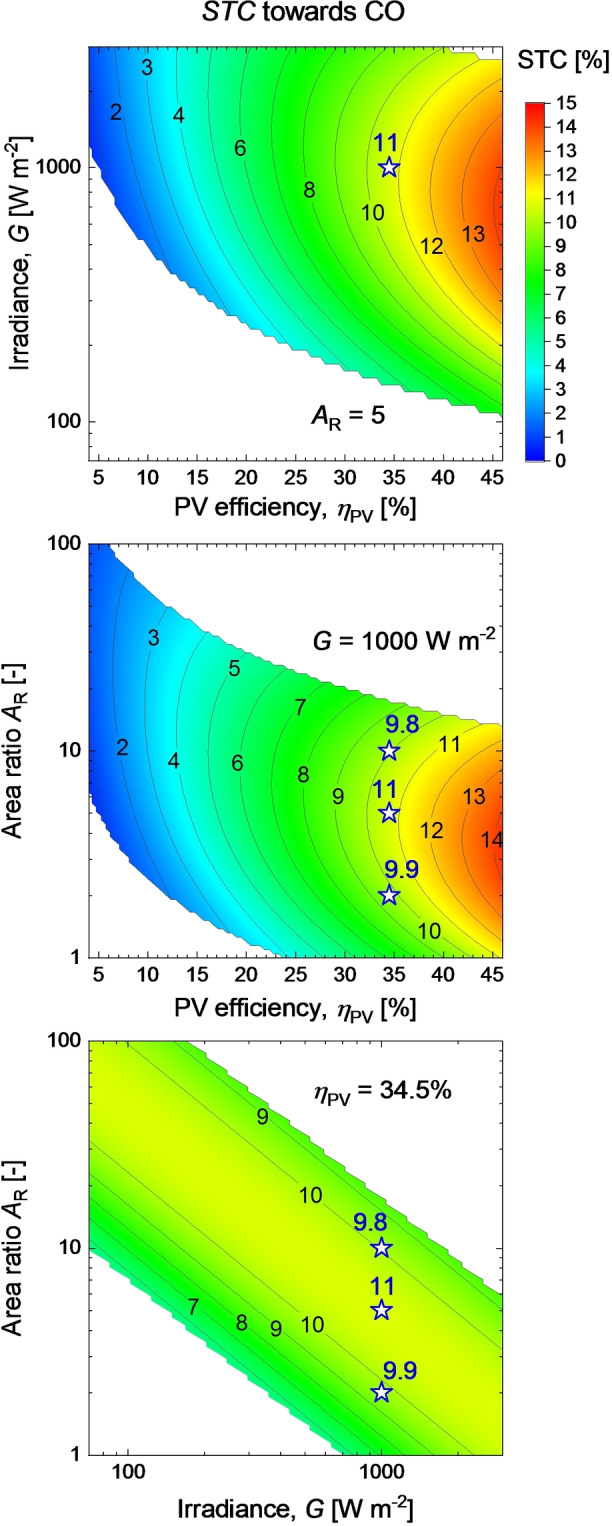
Contour plots of three‐dimensional dependencies of (a) *STC* on *η*_PV_ and *G* at constant *A*_R_, (b) *STC* on *η*_PV_ and *A*_R_ at constant *G*, and (c) *STC* on *G* and *A*_R_ at constant *η*_PV_. Results of the PV‐EC experiment are indicated by stars with *STC* values next to symbols.

In total, there are three distinct combinations for the three‐dimensional slices to be calculated: *STC*(*η*_PV_, *G*) at constant *A*_R_, *STC*(*η*_PV_, *A*_R_) at constant *G*, and *STC*(*G*, *A*_R_) at constant *η*_PV_. These dependencies are presented as contour plots with color‐coded *STC* in Figure 5. For the sake of presentation brevity, we focus on the main product of the electrolyzer, CO. The constants in each case were set to be consistent with those in the PV‐EC experiment, and the data are presented in Figure 4. The results of the PV‐EC experiment are presented as stars, accompanied by the corresponding values of *STC* in Figure 5.

Compared to the two‐dimensional dependencies depicted in Figure 4, the three‐dimensional graphs in Figure 5 offer a more comprehensive overview of the EC potential as a part of a PV‐EC system. This is of particular interest given the variations in irradiance and other system parameters such as PV efficiency in real application scenarios.

One noteworthy aspect of the maps in Figure 5 is the white areas where *STC* values are absent. These white areas represent combinations of parameters that result in the electrolyzer power being outside its characterized operating range. Systems with these parameter combinations supply either too low or too high electric power to EC. Consequently, the areas of implausible or impractical (*η*_PV_, *G*, *A*_R_) combinations are clearly visible in Figure 5.

The dependence of *STC* on *η*_PV_ and *G* at *A*_R_=5 is presented in Figure 5(a). At this specific area ratio, the maximal EC performance is attained at an irradiance close to one sun if the PV efficiency is very high (*η*_PV_>30 %). In Figure 5(a), we can see very close agreement between the map value and the result of the PV‐EC experiment with *STC*=11 % obtained at *η*_PV_=34.5 % and *G*=1000 W m^−2^. Under the irradiance below 1 sun, *STC* towards CO will drop and the electrolyzer will be out of its operating range at *G*<150 W m^−2^, even with the highest PV efficiency. When considering the more common case of *η*_PV_=18–22 % the peak of *STC* as well as the lower edge of the operating region are both located at irradiance levels too high to be practically feasible – approx. 1500 W m^−2^ and 250 W m^−2^ respectively. The position of the operating region and the maximum of *STC* with respect to irradiance can be adjusted with the area ratio in the system.

The interplay between the area ratio and PV efficiency at *G*=1000 W m^−2^ is presented in the next graph in Figure 5(b). The graph enables the identification of the area ratios that are favorable for the electrolyzer in combination with different PV devices under the irradiance of choice. For the characterized CO_2_RR cell, we found that with typical 20 % efficient PV modules, the appropriate range of *A*_R_ is 10–20, while at higher PV efficiencies, the optimal *A*_R_ range shifts down to approx. 4–6. The map in Figure 5(b) predicts that the maximum of *STC* under one sun occurs at *A*_R_ ≈5, with *η*_PV_=34.5 %. This prediction is confirmed by our PV‐EC experiment. The stars in Figure 5(b) indicate the positions of the experimental conditions, while the numbers next to the stars indicate the *STC* values obtained in the PV‐EC experiment.

The effects of irradiance and area ratio at fixed PV efficiency are shown in Figure 5(c). The results of the PV‐EC experiment are in good agreement with the predictions. Under one sun, both predictions and experiment show *STC* maximum for *A*_R_ of approx. 5 for 34.5 % efficient PV device. Now, consider the expected natural variations in irradiance. In most terrestrial scenarios, the irradiance *G* will vary between zero and approximately one sun. In Figure 5(c), the variations in irradiance mean horizontal shifts mostly to the left of the stars marking the one sun PV‐EC experiment. So, for example at *A*_R_=10, the system would remain within the EC operating range at any meaningful irradiance level. For *A*_R_=5, which brings the highest *STC* under 1 sun, the system will critically underpower the EC device at irradiance below approximately 150 W m^−2^. Finally, the system with *A*_R_=2 will be operational at irradiance close to one sun but will be out of operation already under irradiance below approx. 500 W m^−2^. This is remarkable because, in many cases, laboratory‐scale systems and concepts are designed to operate at ratios close to *A*_R_=1.[^17^, ^18^, ^19^, ^20^, ^21^] We can see in Figure 5(c) that for the studied CO_2_RR electrolyzer, the area ratio of 1 is not only inefficient under 1 sun but is also not feasible when irradiance is lower.

## Discussion

The mathematically simple unfolding of the EC characteristics into the multidimensional space of PV‐EC system parameters leads to a surprisingly comprehensive overview of the EC potential in combination with PV of any size and efficiency under any irradiance. The procedure was designed to use a minimal number of key system parameters. Consequently, some parameters are nested within the more general parameters. For example, the effect of the PV temperature is included in the PV efficiency, and the effect of different power coupling components is enveloped in the coupling efficiency. It should be emphasized that all the dependencies presented in this paper are specific to a particular electrolyzer in a particular state. Any variations in the EC characteristics will inevitably change the *STC* dependencies and maps. This property can be employed to evaluate how variations in any EC parameter are projected onto its *STC* potential. By comparing the *STC* potential in different states, electrolyzer operating temperatures, electrolyte molarity, catalyst type or loading, catalyst degradation/conditioning, electrolyzer design parameters, etc., the variations of these parameters can be systematically evaluated in terms of *STC*. Dependencies for the STC limit are useful for loss analysis in already tested PV‐EC systems, as well as for system comparison.

Three‐dimensional *STC* maps are particularly valuable for the primary optimization of a PV‐EC system, especially for guidance on possible variations in operating conditions. Statistical data on irradiance and PV efficiency can be overlaid on the maps to evaluate the expected operating range and maximum PV‐EC power.

Deeper studies of more realistic PV‐EC systems can be performed with appropriate treatment of the coupling efficiency. The coupling efficiency is present in the entire analysis and can be set to a certain average constant value. In more complex cases, a dependence of the coupling efficiency on the PV or EC power can be applied, for example, to account for the typical efficiency profile of a DC‐DC converter. In general, the logic and equations involved in the reverse analysis are based on power balance and are therefore invariant to the PV‐EC coupling mode. Whether it is direct coupling or power conditioning electronics, such as DC‐DC converters or multi‐stage DC‐AC‐DC coupling, the method is valid provided that the coupling efficiency *η*_C_ is properly treated.

Similarly, reverse analysis can be used to analyze any device with known IV characteristics connected to PV systems, particularly in the case of nonlinear devices that are difficult to simulate.

The method allows rapid multiparameter optimization of photovoltaic‐driven electrochemistry devices without numerical modelling. We believe that the method will help to streamline development and minimize the efforts of both photovoltaic and electrochemical communities in the development of highly efficient PV‐EC solutions for sustainable carbon‐neutral energy systems.

## Conclusions

We developed a mathematically simple generic method for rapid evaluation of the solar‐to‐chemical efficiency attainable by the electrolyzer in any PV‐EC system. Practical application of the reverse analysis is demonstrated via processing of the characteristics of the CO_2_RR electrolyzer with CO as the main target product. The results of the analysis are successfully verified with a separate set of experiments where the same EC device is direct‐couped to the 34.5 % efficient PV device under one sun at three different PV‐to‐EC area ratios.

We show that the simple unfolding of the EC characteristics, which can be easily implemented into any spreadsheet software for routine evaluation, provides a comprehensive overview of the EC potential in terms of solar‐to‐chemical efficiency in combination with any PV device under any irradiance with any PV‐to‐EC area ratio. This allows prediction of the electrolyzer performance, primary PV‐EC system optimization, loss analysis, and assessment of electrolyzers in terms of *STC* prior to construction of PV‐EC devices.

The method is based on power balance and can therefore be applied to both PV‐EC systems with direct coupling and those coupled with power electronics.

We believe that the method will help to streamline development and minimize the efforts of both photovoltaic and electrochemical communities in the development of highly efficient PV‐EC solutions for sustainable carbon‐neutral energy systems in the future.

## Nomenclature

PV: photovoltaic cell or module, photovoltaic device
EC: electrochemical cell or stack, electrochemical device
PV‐EC: combined device with PV connected to EC coupled directly or via voltage converter
CO_2_RR: carbon dioxide reduction reaction
*V*
: voltage
*J*
: current density
JV: current density – voltage characteristics
*J*_EC_(*V*): input JV of an EC device
*J*_p_(*V*): output partial JV of an EC device for one product
*J*_CO_(*V*): output JV for CO production
*J*_H2_(*V*): output JV for H_2_ production
*E* ^°^_p_ [V]: reference potential for the product in question
*E* ^°^_CO_ [V]: reference potential for CO 1.34 V
*E* ^°^_H2_ [V]: reference potential for H_2_ 1.23 V
*G* [Wm^−2^]: plane of array irradiance, power density of light arriving at the PV device
*P* [Wm^−2^]: in this work all quantities designated as “*P*” with subscripts refer to power densities
*P*_mpp_ [Wm^−2^]: maximum output power density of a PV device.
*P*_EC_ [Wm^−2^]: input electrical power density of an EC device.
*P*_p_ [Wm^−2^]: output power density of one EC product.
*P*_CO_ [Wm^−2^]: output power density of CO production.
*P*_H2_ [Wm^−2^]: output power density of H_2_ production.
*A*_PV_ [m^2^]: geometric aperture area of the PV device (aperture area of the concentrator lens or mirror array for the case of concentrator PV) receiving plane of array irradiance *G*.
*A*_EC_ [m^2^]: total geometric active area of an EC device (for an EC stack–sum of the areas of all cells).
*A*_R_ [−]: the ratio of the PV area *A*_PV_ to the EC area *A*_EC_.
*η*_PV_ [%]: power conversion efficiency of a PV device
*η*_C_ [%]: coupling efficiency in PV‐EC system, 100 % for ideally coupled system
*η*_EC_ [%]: power conversion efficiency of an EC cell
*STC* [%]: solar‐to‐chemical efficiency, end‐to‐end power conversion efficiency of a PV‐EC system

## Experimental SectionElectrochemical Cell and CO_2_RR Setup

**Figure 6:**
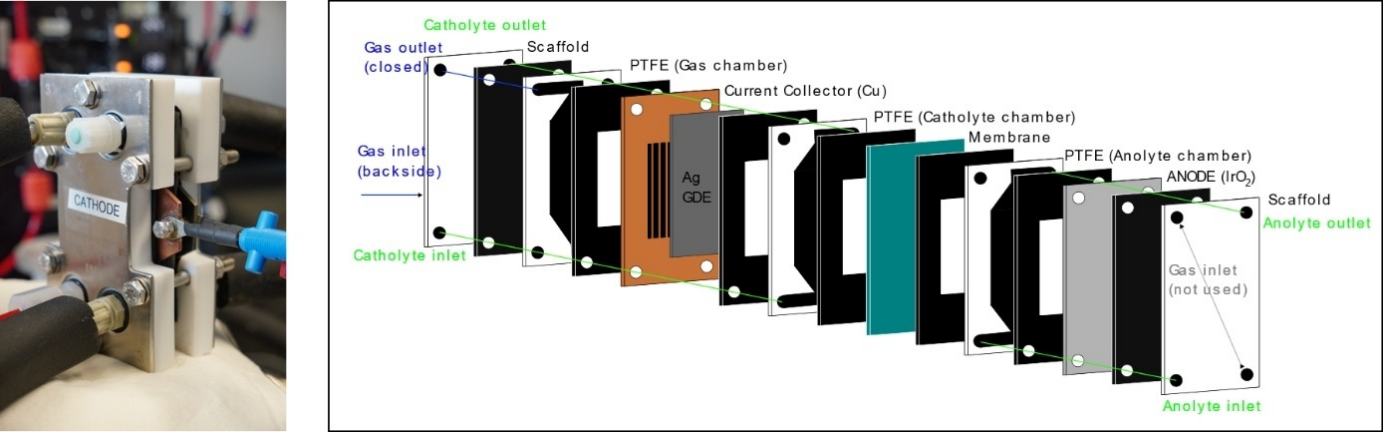
Picture of electrochemical cell (left) and its exploded schematic (right).

We performed the CO_2_RR in a microflow electrochemical cell (Electrocell) consisting of a sequence of plates equipped with dedicated inlet and outlet paths for the electrolyte and gases, as shown in Figure 6.

**Figure 7:**
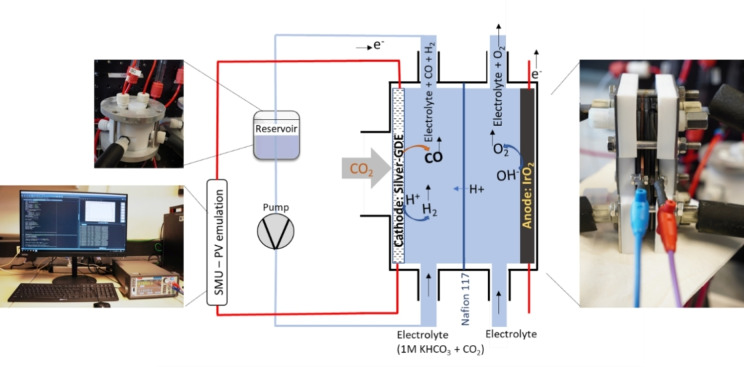
Schematic of the experiment setup highlighting catholyte reservoir, PV emulation system and electrically connected EC.

The anode was an IrO_2_ bulk plate provided by the electrochemical cell manufacturer. The cathode was a silver gas diffusion electrode, as described in the following section. Both electrodes had a geometric active area of 8.8 cm^2^. The electrochemical cell was equipped with a Nafion 117 cation exchange membrane. CO_2_ was purged independently in the anolyte and catholyte reservoirs at a flow rate of 60 sccm. Additionally, CO_2_ was directly purged in the cathode compartment of the EC cell through the microporous layer of the gas diffusion electrode (GDE) at a flow rate of 40 sccm. Both the catholyte and anolyte were CO_2_ saturated 1 mol L^−1^ KHCO_3_ solutions continuously pumped at a flow of 150 mlmin^−1^. The two electrolyte reservoirs were airtight Teflon containers with a volume capacity of approximately 100 mL, equipped with liquid inlet and outlet connections, gas inlet and outlet connections, and a built‐in temperature sensor. From the reservoirs to each compartment of the electrochemical cell, the electrolyte pipes were continuously and externally heated at 45 °C during the CO_2_RR. Schematic of the CO_2_RR experiment is shown in Figure 7

## Preparation of the Catalyst

A commercial silver powder catalyst (IoLiTec, 50–60 nm, 99.9 %) was added to a mixture of water (MilliporeSigma, Mili‐Q®, IQ7000, 18.2 MΩ.cm), propanol (Sigma Aldrich, AR), and Nafion (Chemours, D521CS. The obtained catalyst ink was homogenized using an ultrasonic bath for 30 min.

Silver‐based gas diffusion electrodes (GDE) were fabricated by airbrushing the silver ink onto Freudenberg (H23C2) carbon papers until a load of 1 mg cm^−2^±0.1 mg cm^−2^. During the deposition, we simultaneously heated the carbon papers at 70 °C to allow faster evaporation of the solvents.

## CO_2_RR Experiment and *JV* Investigation of EC

We connected EC to a potentiostat (OrigaLys OGF 05 A) and successively increased the voltage from 2.4 V to 4.4 V with 100 mV steps. The system was maintained at each voltage for 7 min, and a resting time (applied voltage OFF) of 4 min was imposed between each voltage. The resulting current values were measured and recorded every 100 ms by the potentiostat, while the products were quantified by online gas chromatography (Shimadzu GC 30301 – BID detector).

## EC Characteristics and Smoothing Procedure

**Figure 8:**
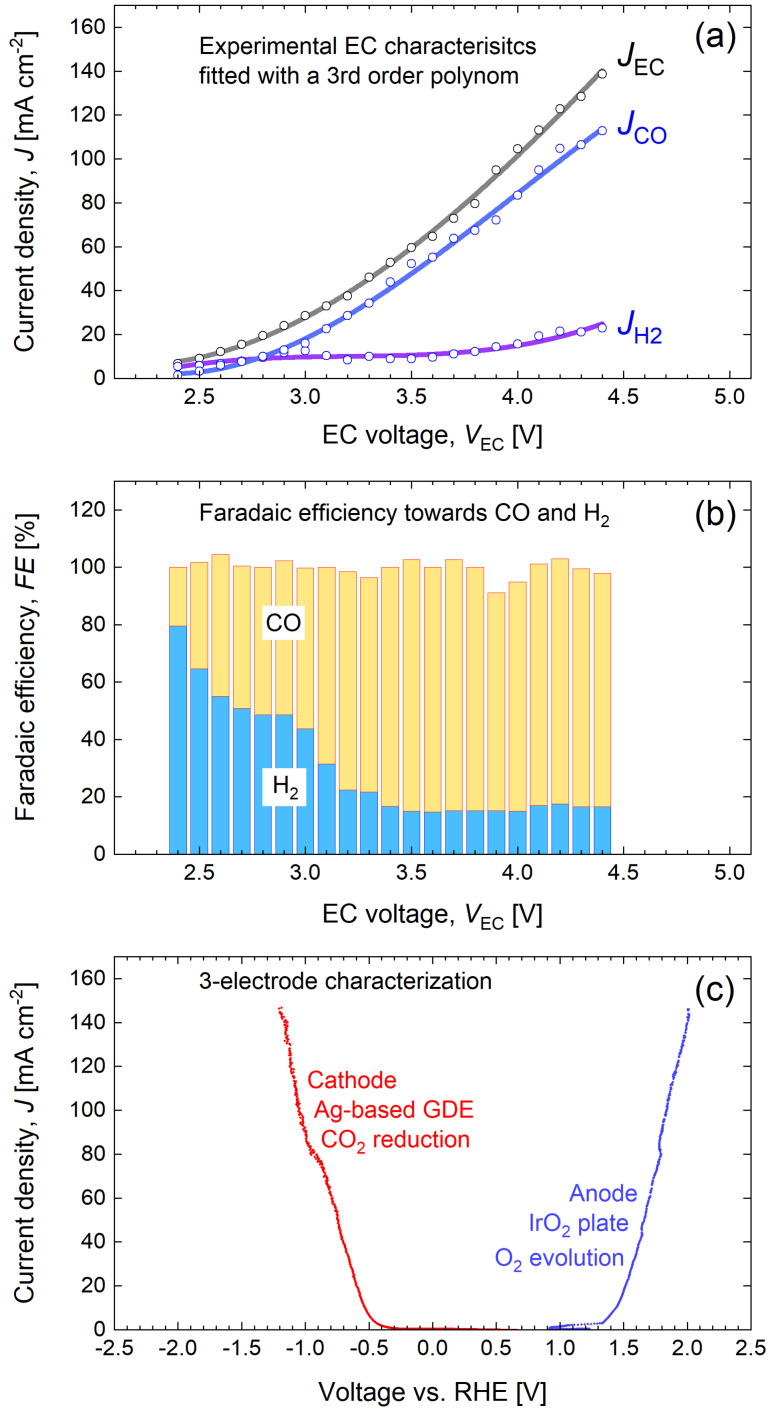
(a) The original measured EC characteristics: input current density *J*_EC_(V), output CO current density *J*_CO_(*V*), output H_2_ current density *J*_H2_(*V*) and the results of the smoothing procedure with a third‐order polynomial fit. (b) Stacked bar diagram of the Faradaic efficiency towards CO and H_2_ (c) 3‐electrode LSV measurements for the cathode and anode half reactions.

Detailed measurement of the input and output *JV* characteristics of the EC cell, including product characterization, is a relatively long experimental procedure with related run‐to‐run scatter in measurement points. This scatter is translated into the results of the reverse analysis and maps presented in this paper. To focus on the main trends and to improve the visual perception of the analysis, we smoothed the measured *J*_EC_(*V*), *J*_CO_(*V*), and *J*_H2_(*V*) dependencies with third‐order polynomial fit. The original data points and the results of the fitting are presented in Figure 8(a).

The Faradaic efficiency (FE) towards CO and H_2_ is plotted as a stacked bar diagram in Figure 8(b). The cell showed a consistently high total FE of close to 100 % over the entire voltage range measured. At the beginning of the voltage range, the cell produces more hydrogen than CO, but as the voltage increases, hydrogen production is suppressed down to FE of approx. 20 %. Conversely, CO production is favored at higher EC voltages, making CO the dominant product of the reaction with FE of approx. 80 %. at *V*_EC_ above 3.1 V

Linear sweep voltammetry (LSV) curves were measured with a potentiostat (OrigaLys OGF 05 A), scanning potentials from 0 to 4.5 V at a rate of 5 mV s^−1^, in a CO₂‐saturated 1 M KHCO₃ electrolyte (pH 7.5). Simultaneously, the respective potentials of the silver‐based cathode and the working electrode were measured and recorded with a data logger (Graphitec midi logger GL240) with respect to a reversible hydrogen electrode RHE (Hyfroflex), which was used as the reference electrode. The RHE was positioned parallel to each electrode in the respective reaction chamber. The results of the 3‐electrode LSV measurements for the CO_2_ reduction half‐reaction at the Ag‐GDE cathode and the oxygen evolution half‐reaction at the IrO_2_‐anode are presented in Figure 8(c). The GDE cathode shows good performance with an onset potential of approx. −0.34 V vs. RHE, which is in the middle of the range reported in the literature.[^58^, ^59^]

## PV‐EC Experiment

**Figure 9:**
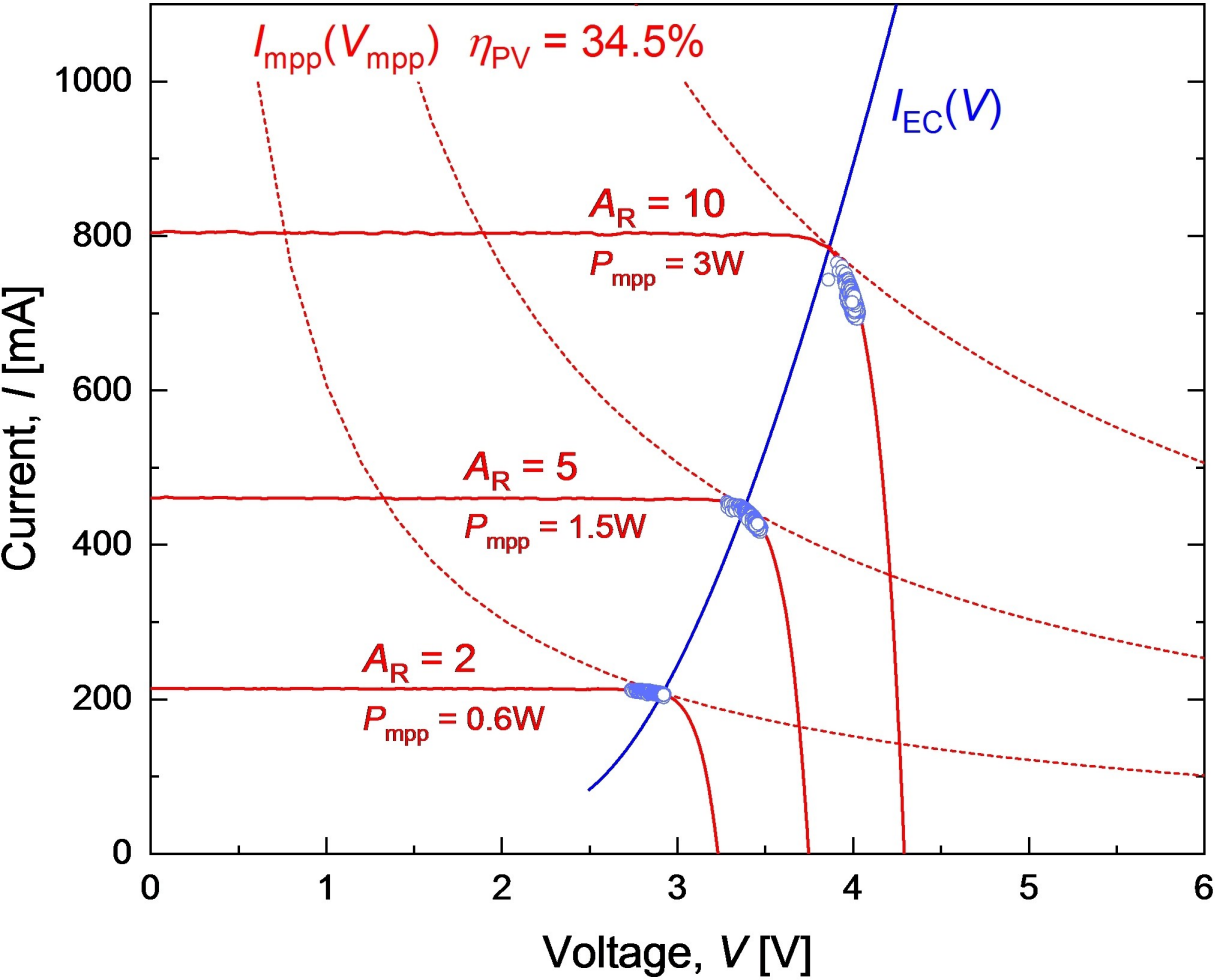
EC characteristics *J*_EC_(V), *I*_mpp_(*V*_mpp_) lines used to find points of optimal PV‐EC coupling, and PV IVs of GaAs PV device scaled for optimal coupling at three area ratios of 2, 5 and 10. Points represent working points during three PV‐EC experiments performed with emulated PV IVs.

Validation PV‐EC experiments were performed with the EC cell direct‐coupled to a custom in‐house developed PV emulator, which reproduces the required PV current voltage characteristics with precision and accuracy on par with AAA solar simulator.^[60]^ The emulation approach was selected to achieve the necessary flexibility and accuracy in PV scaling, thus ensuring proper coupling to the small‐scale laboratory EC device at different area ratios. The IV characteristics of the GaAs PV cell measured under concentrated light were resized to emulate PV devices with the required area ratios of 2, 5, and 10 and optimal coupling to EC. During the PV IV adjustment, the EC area *A*_EC_=8.8 cm^2^ is multiplied by the required *A*_R_ to determine the necessary value of *A*_PV_ in each case. Subsequently, the area and maximum power of the measured PV cell were used to calculate the PV *P*_mpp_ required to conduct the experiment with each particular *A*_R_. The *P*_mpp_ values were used to determine the points of optimal coupling of the PV device to the EC device in question. This is accomplished by drawing *I*_mpp_(*V*_mpp_) dependencies for the required *P*_mpp_ values, as discussed in the literature[^46^, ^51^, ^61^] and illustrated in Figure 9. The intersections of the *I*_mpp_(*V*_mpp_) curves with the electrolyzer characteristics determine the points of optimal coupling for each *A*_R_. The current and voltage of the PV IV were scaled to achieve the optimal matching in each case as illustrated in Figure 9. The rationale for this free scaling is that, in practice, both PV and EC devices will be presented by modules and stacks encompassing numerous PV and EC cells on each side. Consequently, different combinations of individual cell areas and numbers of serially connected cells permit a relatively smooth adjustment of both current and voltage in the system.[^1^, ^32^, ^49^, ^52^] After the preparation of PV IV, the EC was connected to a SMU (source measure unit Keithley 23060) controlled by a computer algorithm emulating the respective IVs of the 34.5 % efficiency GaAs PV device. The working points during the three PV‐EC experiments, recorded every 100 ms, are shown in Figure 9. The products were quantified using gas chromatography.

## Supplementary Information Summary

MS EXCEL spreadsheet encompassing the analysis routine applied to the dataset presented in this paper (Version 5) is available at Zenodo https://doi.org/10.5281/zenodo.14165599

## Author Contributions

Oleksandr Astakhov: conceptualization, investigation, data curation, formal analysis, visualization, writing – original draft, writing – review & editing.

Thérèse Cibaka: investigation, data curation, writing – review & editing.

Lars Wieprecht: investigation, data curation.

Uwe Rau: supervision, formal analysis.

Tsvetelina Merdzhanova: conceptualization, supervision, data curation, formal analysis, writing – review & editing.

## Conflict of Interests

The authors declare no competing interests.

1

## Supporting information

As a service to our authors and readers, this journal provides supporting information supplied by the authors. Such materials are peer reviewed and may be re‐organized for online delivery, but are not copy‐edited or typeset. Technical support issues arising from supporting information (other than missing files) should be addressed to the authors.

Supporting Information

## Data Availability

The data that support the findings of this study are available in the supplementary material of this article.
