# Supplementary material for: Unfolding Electrolyzer Characteristics to Reveal Solar‐to‐Chemical Efficiency Potential: Rapid Analysis Method Bridging Electrochemistry and Photovoltaics
Source: ChemSusChem. 2024 Nov 26;18(7):e202402027. doi: 10.1002/cssc.202402027 (PMC11960588; doi:10.1002/cssc.202402027)
Supplement: Supplementary file 1 — Supporting Information [file CSSC-18-e202402027-s001.pdf]

ChemSusChem

Supporting Information

Unfolding Electrolyzer Characteristics to Reveal Solar-to-Chemical Efficiency Potential: Rapid Analysis Method Bridging Electrochemistry and Photovoltaics

Oleksandr Astakhov,* Thérèse Cibaka, Lars Wieprecht, Uwe Rau, and Tsvetelina Merdzhanova

Unfolding Electrolyzer Characteristics to Reveal Solar-to-Chemical Efficiency Potential: Rapid Analysis Method Bridging Electrochemistry and Photovoltaics

Oleksandr Astakhov[*1], Thérèse Cibaka[1], Lars Wieprecht[1], Uwe Rau[1,2], Tsvetelina Merdzhanova[1]

[1] Photovoltaics (IMD-3), Forschungszentrum Jülich GmbH, 52425 Jülich, Germany

[2] Faculty of Electrical Engineering and Information Technology, RWTH Aachen University, Mies-van-der-Rohe-Straße 15, 52074 Aachen, Germany

[*]Corresponding author Oleksandr Astakhov E-mail: o.astakhov@fz-juelich.de

Supplementary information

The analysis method presented in the paper is applied to the characteristics of an experimental CO2 reduction reaction (CO2RR) EC cell with Ag catalyst, which produces CO as the main product and H2 as a byproduct. The MS EXCEL spreadsheet encompassing the analysis routine applied to the dataset presented in the paper is available at Zenodo https://doi.org/10.5281/zenodo.14165599
